# Mitochondrial responses to extreme environments: insights from metabolomics

**DOI:** 10.1186/s13728-015-0026-9

**Published:** 2015-05-04

**Authors:** Katie A O’Brien, Julian L Griffin, Andrew J Murray, Lindsay M Edwards

**Affiliations:** Centre of Human & Aerospace Physiological Sciences, King’s College London, London, UK; MRC Human Nutrition Research Unit, Elsie Widdowson Laboratory, Fulbourn Road, Cambridge, England; Department of Biochemistry, University of Cambridge, Tennis Court Road, Cambridge, UK; Department of Physiology, Development and Neuroscience, University of Cambridge, Downing Street, Cambridge, UK; Fibrosis Drug Performance Unit, GlaxoSmithKline Medicines Research Centre, Stevenage, UK

**Keywords:** Metabolomics, Extreme environments, Mitochondria

## Abstract

Humans are capable of survival in a remarkable range of environments, including the extremes of temperature and altitude as well as zero gravity. Investigation into physiological function in response to such environmental stresses may help further our understanding of human (patho-) physiology both at a systems level and in certain disease states, making it a highly relevant field of study. This review focuses on the application of metabolomics in assessing acclimatisation to these states, particularly the insights this approach can provide into mitochondrial function. It includes an overview of metabolomics and the associated analytical tools and also suggests future avenues of research.

## Review

### Background

Human populations have successfully settled in an extraordinarily diverse range of habitats, many of which present significant environmental challenges to life including the extremes of temperature or altitude. Recent research has highlighted the role that natural selection is playing in shaping the genomes of humans in such niche environments [1-4]. However, despite significant promise, technical advances in other system-wide disciplines such as proteomics and metabolomics have yet to yield widespread insights into human acclimatisation and adaptation to extreme environments. We have previously put forward the notion that systems biology and the study of humans in extreme environments are natural symbionts [5,6]. In this review, we highlight the application of metabolomics in such studies, with three specific aims:

1. To serve as a broad introduction to the field of metabolomics for the non-expert, with the aim of promoting its more widespread use in the field.
2. To illustrate how metabolomics can provide insight into the role of mitochondria in acclimatisation to extreme environments.
3. To suggest potentially fruitful avenues for future research, deploying metabolomics methods in human extreme environmental research.

Broadly, the review will follow these points in order, starting with an overview of the methods of metabolomics.

#### A brief introduction to metabolomics

In recent years, technological advances have allowed for the unbiased detection, identification and semi-quantification of many low molecular weight (<1,500 Da) compounds in cells, tissues, biofluids or organisms, in a single experiment. Metabolites are the reactants, intermediates or products of enzymatic reactions in the body. They represent the final products of cellular processes including the activity of mRNA and proteins and are key components of mitochondrial processes such as the tricarboxylic acid (TCA) cycle and β-oxidation. Investigation into the metabolic phenotype, or metabolome, in response to a physiological stimulus or genetic modification, termed metabolomics, is a functional level of systems biology [7]. Unlike changes in the proteome or transcriptome, which are usually measured over minutes to hours, the metabolome is highly dynamic and subject to fluxes over a period of seconds or less. The metabolome is therefore an extremely sensitive measure of biological phenotype and can unmask seemingly silent phenotypic changes that have no frank physiological or characteristic behaviours [8,9], particularly when used with other -omic approaches [10-12].

#### Analytical tools

The human metabolome is estimated to comprise of many tens of thousands of individual metabolites, including both those confirmed and predicted, endogenous and xenobiotic [13-15]. The human metabolome database (available at www.hmdb.ca), for instance, contains 41,992 metabolite entries.

The atomic arrangement of metabolites is highly diverse. Chemical properties vary enormously, ranging hydrophilic, polar metabolites with a low molecular weight (e.g. amino acids) to hydrophobic, non-polar higher molecular weight metabolites (e.g. lipids) [8]. This sets metabolome analysis apart from that of the transcriptome or proteome as information within DNA, RNA or peptides is encoded in patterns constructed from uniform constituent chemical subunits (i.e. polymerised nucleic acids and amino acids). Moreover, metabolite concentrations also vary from the mmol/l to pmol/l range.

This extreme diversity makes unbiased detection (as is possible in transcriptomics) exceptionally challenging. Indeed, detection and quantification of all metabolites in human samples cannot currently be achieved using a singular analytical technique. Furthermore, there are few good methods for amplification of metabolite levels as there exists for genomics and transcriptomics in the various forms of the polymerase chain reaction. However, nuclear magnetic resonance (NMR) spectroscopy and mass spectrometry (MS) coupled with chromatography are both popular and increasingly used analytical approaches [13]. The resulting signals are identified using data libraries, such as the human metabolome database [16], or experimentally using analytical chemistry techniques for structure elucidation and with metabolite standards where available.

#### Nuclear magnetic resonance spectroscopy

NMR experiments are performed on atomic nuclei with a non-zero spin quantum number (a quantum property of the nucleus related to angular momentum and charge and often symbolised as a spinning magnetic field), such as ^1^H, ^13^C or ^31^P. All of the aforementioned nuclei possess a spin angular momentum quantum number ½ and can exist in two energy levels—often referred to as spin up or spin down.

In a large population of identical nuclei at equilibrium, the spin and related magnetic moments of individual nuclei have equal energy (i.e. they are degenerate), and both are randomly arranged. In an NMR experiment, nuclei are exposed to a powerful magnetic field which creates a population difference between the spin up and down nuclei as there is a slight excess of the lower energy state (this is often the spin down state). The energy difference is relatively small, comparable to the thermal energy in the system, and irradiation with a pulse of radiofrequency wave of the right frequency will convert spins of the lower energy state to the higher one until the population difference is cancelled out. The irradiating radiofrequency waves are then turned off, allowing the nuclei to relax back to their equilibrium potential over a period of time by emitting a characteristic radiofrequency signal. This is called a free induction decay, and if the signal is transferred from time to frequency using a mathematical approach called a Fourier transform, then a spectrum arises. The area of the resonance is directly proportional to the concentration of nuclei that generate it, making NMR innately quantitative. As described, this would be a poor analytical technique, only able to separate out nuclei of different atoms, but the chemical environment each nucleus is found within modulates the frequency. Thus, the spectrum produced by a metabolite will encode information about the structure making it a powerful analytical approach. Another important advantage is that the sample itself has only been exposed to a magnetic field and radiofrequency pulse, making NMR completely non-destructive enabling re-use of samples and avoiding the instrument being impaired by the accumulation of unwanted analyte [17].

As described above, the properties of the chemical environment surrounding the nuclei cause a shift in the resulting resonances. This is caused by a number of factors, including the shielding effects of the electron cloud surrounding the nucleus that alters the local field at the nucleus. The ^1^H nuclei resonances will reflect the chemical group to which that particular nucleus belongs. Variation in this NMR frequency, or ‘chemical shift’, is small (most ^1^H frequencies only vary within a range of 10 parts per million) but can be measured with great accuracy enabling a detailed identification of compounds [17,18].

Chemical shift is also affected by the properties of surrounding nuclei. The energies of nuclei positioned close together interact, a phenomenon denoted spin-spin coupling, which causes the chemical shift to be split into sub-peaks. Distinct molecules have a characteristic number and pattern of peaks and sub-peaks [18], and this can be used to great effect in two- and multi-dimensional NMR spectroscopy [19].

Analysis of NMR spectra gives precise information regarding chemical structure and abundance of the molecules of which the nuclei form a part, thus enabling metabolite identification [17,18]. The quantitative nature, high reproducibility and relatively simple sample preparation make NMR a highly desirable technique. It is also non-selective, as the sensitivity is independent of the hydrophobicity or acidity of the compounds being analysed [8]. One large downside to the approach is its relatively poor sensitivity, which arises because the nuclear transitions that are measured in the NMR experiment are very close to the thermal energy of the system, meaning population differences between the low and high energy states are very small [8]. Only those metabolites in high abundance (100 nmol/l to 1 μlmol/l or higher) can be detected, with usually less than 100 metabolites detectable per sample in a typical liquid-state metabolomics experiment [18,20]. Further, 1D spectroscopy (typically used for metabolomics experiments) results in spectral crowding: alterations in low-abundance metabolites may be obscured by those species in higher abundance and with similar chemical shifts, which in turn limits biomarker discovery. Although 2D NMR spectroscopy offers a solution to this problem, it also results in increased instrument time and, therefore, cost. However, this is beginning to be addressed through developments in cryoprobes to improve sensitivity and sparse sampling to speed up 2D acquisitions [21-23].

NMR experiments are typically performed on samples in the liquid state. This includes biofluids, such as plasma, serum or urine, and also metabolites extracted from tissue that are re-dissolved in solvent. This is because in the liquid state, metabolites tumble readily. The action of tumbling ensures that a narrow NMR resonance is obtained which simplifies the resultant spectra. The spectra produced from NMR analysis of tissue extracts are generally well resolved, and valuable information can be gained from this. However, as the integrity of the tissue is destroyed during the extraction process, this is not necessarily a good representation of *in vivo* function. Another option is to use untreated intact tissue in the solid state. High-quality, well-resolved NMR data can be achieved from this matrix using high-resolution magic angle spinning (HR-MAS). The ‘magic angle’, 54.7° relative to the applied magnetic field, reduces NMR peak widths, attenuating the line-broadening effects produced in solid samples and so improving spectral quality to near-liquid state [24,25]. The information acquired from HR-MAS is therefore more comparable to *in vivo* function. It also enables the study of time-dependent metabolic processes to be assessed in a tissue [26].

Finally, *in vivo* NMR analysis of tissue biochemistry can be conducted using a surface radiofrequency coil, which allows for the detection of compounds in localised regions adjacent to the coil (and hence the sample surface) [27]. In techniques such as 31-phosphorus magnetic resonance spectroscopy (^31^P-NMR), this enables detection of tissue phosphorus metabolites and phosphocreatine and so provides insight into tissue energetics [28].

#### Mass spectrometry

The other major technique currently applied in metabolomic analysis is MS. This operates by the formation of positively or negatively charged species (ions) from analytes of interest (in this case metabolites), which are then separated according to their mass-to-charge ratio.

MS is performed either with or without a precedent separation step. While gas chromatography-MS (GC-MS) was the dominant MS platform for some years, improvements in technologies have led to the widespread adoption of liquid chromatography-MS (LC-MS) as the analytical tool of choice for MS metabolomics. This includes technologies that enhance sensitivity and separation resolution, such as ultra-performance liquid chromatography [8]. However, there are many applications where GC-MS is still very popular (e.g. analysis of total fatty acids, analysis of core metabolism in plants). The high sensitivity afforded by LC-MS has greatly aided in the development of metabolomics, enabling detection of hundreds or even thousands of metabolites in a given sample [29]. This includes detection of metabolites at very low abundance (1 pmol/l) [8]. In spite of this, metabolite identification remains a significant hurdle.

#### Chromatography

Chromatographic separation of the molecular species within a sample is usually used prior to infusion into the mass spectrometer. This simplifies the resultant mass spectra collected and also improves the ionisation of individual analytes. The most commonly used techniques are liquid or gas chromatography (LC and GC, respectively).

In LC, the analyte mixture is dissolved into a liquid mobile solvent (the mobile phase), which is then passed through a column containing surfaces coated with specific interaction chemistries (the stationary phase). The speed at which individual analytes pass through the column is dependent upon their physiochemical interaction with the stationary phase. As analytes are separated in the liquid phase, this makes it suitable for analysis of both polar and lipophilic thermally labile compounds in the solution. The reduction in pre-processing in comparison to GC (described below) explains one of the reasons for its increased popularity. Different LC column chemistries enable efficient separation of metabolites with varying properties. Non-polar columns with carbon chain chemistries, such as the near-ubiquitous C_18_ column, enable efficient separation of lipid species, whereas the polar chemistries found within hydrophobic interaction chromatography columns (HILIC) enable separation of more polar compounds [8,20].

By contrast, in GC, analytes are vaporised and so the mobile phase is gaseous. The analyte species therefore need to be non-polar volatiles, meaning a volatile derivatisation step is usually required. For instance, fatty acids are derivatised to form fatty acid methyl esters and polar head groups are often reacted with trimethylsilyl derivatives. In GC, the temperature of the column is increased in an oven, allowing a partition of metabolites between the stationary and mobile phases at different temperatures. Thus, a chromatogram is produced according to a temperature gradient.

#### Ionisation

Components eluting from the chromatographic column are introduced to the mass spectrometer via a special ionisation interface. The ionisation technique adopted is dependent upon the prior chromatography step. Electron ionisation is used almost exclusively with GC as it requires volatile analytes. It is a very reproducible form of ionisation, and this has aided the production of GC-MS libraries of metabolites. It produces inherent molecular fragmentation, which can be applied for metabolite identification.

Electrospray ionisation on the other hand ionises non-volatiles, making it suited for use with LC and is optimal for the separation of complex biological fluids [30]. Here, ions are formed in solution within the needle before droplet formation from the Taylor cone. Subsequent measurement of metabolite species is dependent upon the ionisation mode. In negative ionisation mode, there is preference for detection of anionic species such as organic acids, whereas in positive ionisation mode, there is preference for detection of neutral (which often pick up a H^+^ or other suitable cation during the process to form an adduct) and cationic metabolites including protonated amino acids and amines.

A downside of this essential step is the possibility of ion suppression, which occurs in complex biological molecular mixtures when analytes compete for charge during the ionisation process [31]. The detected ‘abundance’ signal of a compound can be affected by other substances including analytes and contaminants. Although the initial chromatographic separation helps to reduce suppression effects by simultaneously reducing the number of species entering the ionisation step, the problem can still persist [32].

#### Mass analysis

The fundamental principle of MS is that ions, including molecular, fragment and adducts, are separated according to their mass-to-charge ratio (*m*/*z*). In metabolomics experiments, the vast majority of ions carry a single charge (i.e. *z* = 1), meaning that *m/z* usually = *m.*

Although it is possible to resolve many thousands of signals in a single MS experiment, the unambiguous identification of unique metabolites presents the most significant current analytical and experimental challenge for investigators [33]. Positive identification of a metabolite requires several parameters, including accurate mass, fragmentation pattern, isotope abundance pattern and retention time, to match with that of a purified metabolite under identical conditions. This definitive identification is not plausible for large numbers of metabolites. It is therefore broadly accepted for metabolites to be ‘putatively annotated’, a term defined by the Metabolomics Standards Initiative [34]. This method of identification uses a single measured parameter, such as accurate mass, and matches this to a metabolite present in a library or database. Although less time-consuming than definitive identification, the confidence in correct identification is lower [35], hence ‘key’ compounds still need to be annotated more rigorously. Indeed, some claim that unless metabolites are identified by two orthogonal techniques the assignment should still be labelled as tentative [34].

It is also worthy of note that structural similarities within lipid classes (e.g. the eight broad classes outlined in the LIPID MAPS classification system [36]) allow the measurement and (at least partial) classification of a large number of lipid species in a single MS run. However, the increased structural complexity and size of many lipids, including differing isomers and fatty acid constituents, makes completely unambiguous identification challenging.

#### Targeted and non-targeted metabolomics

Metabolomics can either be targeted or non-targeted. Both have their advantages and disadvantages and can be highly effective and complementary when used in conjunction.

Untargeted metabolomics attempts to measure all the analytes in a sample, including chemical unknowns. In this approach, there is no specific *a priori* hypothesis stating which metabolites are related to the (patho-) physiological change. It aims to produce data on an extensive range of metabolites present in multiple metabolite classes or pathways that are dispersed across the metabolic network. The metabolome coverage is therefore intended to be unbiased and as comprehensive as possible. This method is not quantitative, and metabolite identification is a challenge. In order to reduce the resulting data sets into more manageable entities, dimension reduction techniques such as principal components analysis (PCA) or multidimensional scalings are required. The results of such experiments can be inductive or hypothesis-generating and can provide insight into novel changes occurring to the metabolome as a result of the perturbed state [8,37].

Targeted metabolomics involves the detection of a specific number of metabolites (typically in the order of tens to hundreds) that are related in function or class. This method is used in hypothesis testing or deduction studies where the metabolites (or at least, pathways) of interest are known.

A major development effort is required to establish a successful targeted technique. Absolute quantitative metabolite concentrations are determined with high specificity and accuracy by using the addition of internal standards. These are typically isotopically labelled versions of the endogenous metabolites, usually containing ^13^C or ^2^H isotopes [8]. As only those targeted metabolites are detected, this does mean that the number of discovery opportunities is reduced. However, one hybrid option is to use semi-quantitative methods where a number of ‘class-specific’ standards are spiked into samples. This relies on the assumption that similar classes of compounds give comparable signals.

### Metabolomics and extreme environments

The metabolomics approach aims to measure metabolites at baseline or in the context of a perturbed state. Yet there are a limited number of ethical physiological perturbations that can be used in experiments on humans; these include exercise, nutrition, some drugs and the environment. It has been argued that environmental physiology can provide insight into the system-level understanding of the human body [5] and into a number of pathological states.

Studying the healthy human response to extreme environments has been used widely to investigate fundamental physiology (perhaps with pathological importance) without the confounding factors and complications prevalent in a diseased population [38]. This is particularly relevant for investigations into high-altitude physiology. Inadequate availability of oxygen to the tissues (hypoxia) as occurs at high altitudes is also a feature of a plethora of clinically important conditions. These include lung disease, heart failure, anaemia, cancer and regional vascular diseases [39-43]; indeed, any clinical condition where either convective or diffusive oxygen transport is impaired.

#### Metabolomics and altitude

Hypoxia at high altitudes results from a reduction in inspired partial pressure of oxygen. Acclimatisation is partly dependent upon changes to oxygen-dependent processes, including mitochondrial oxidative phosphorylation. Oxidative means of energy production are essential for normal physiological function as few cells are able to rely solely upon anaerobic means of energy generation [44]. Severe hypoxia can lead to such a decline in bodily functions that it may rapidly become fatal [45]. Survival in the face of reduced oxygen availability thus requires a profound shift in metabolic processes.

The few extant metabolomics experiments investigating the physiology of high altitude have focused on profiling plasma, serum or urine from hypoxia-exposed individuals. Tissot van Patot and colleagues [46] performed metabolomic profiling on samples taken from subjects following 8-h exposure to 12% oxygen (equivalent to ~4,300 m) in a hypobaric chamber. Assessment of plasma using ^1^H-NMR revealed an increase in L-lactic acid (HMDB00190) and succinic acid (HMDB00254) concentrations, by 29% and 158%, respectively, in response to hypoxia [46]. These findings are in line with previous studies suggesting that hypoxia prompts a shift towards anaerobic means of energy generation through an active shunting of pyruvate away from entry into the TCA cycle towards lactate production and an inhibition of TCA cycle activity [47-49]. Accumulation of succinate may be indicative of a concerted downregulation of TCA cycle and electron transport chain (ETC.) activity, given that succinate dehydrogenase is also complex II of the ETC. Interestingly, succinate is also suggested to have a toxic effect in the heart in response to ischemia, as its accumulation has been directly linked to mitochondrial reactive oxygen species production from complex 1 [50]. Plasma analysis by Tissot van Patot and colleagues also revealed a reduction in the levels of the antioxidant glutathione. This was coupled with an increase in urinary prostane excretion, as assessed using LC-MS [46]. This supports the concept that oxidative stress increases in response to hypoxia [51,52].

It is not clear whether these apparent adjustments in metabolic processes during hypoxia translate to an altered exercise economy at a whole body level. When considering the energetics of the skeletal muscle at altitude, it seems that exercising metabolites are unaltered in subjects trekking to 5,300 m and climbers ascending above 7,950 m. By the use of ^31^P-NMR, the half-life of phosphocreatine (PCr) (a widely accepted measure of mitochondrial function) was found to remain at sea level values, indicating that skeletal muscle mitochondrial function was maintained. This occurred despite a reduction in muscle cross-sectional area and aerobic capacity. Thus, the metabolic changes occurring in healthy humans at high altitude preserve *in vivo* function in the face of profound structural changes [28] in a manner that remains poorly understood. In the human heart, however, the ratio of PCr/adenosine triphosphate (ATP) becomes impaired [53,54], perhaps suggesting that in the heart, which has a greater mitochondrial density and metabolic rate than the skeletal muscle, oxidative phosphorylation is not preserved.

It appears that, given adequate acclimatisation time, healthy humans are capable of achieving successful metabolic acclimatisation to ameliorate oxidative stress. Placenta extracts analysed after labour birth from subjects either at altitude (3,100 m) or sea level using ^1^H-NMR and ^31^P-NMR spectroscopic analysis [55] suggested that those placentas which had developed at altitude had adapted to hypoxia, demonstrating a blunted oxidative stress response during labour and a preconditioning to energy storage through higher PCr concentrations [55].

#### High-altitude pulmonary oedema

If ascent to high altitudes is rapid with insufficient time allowed for acclimatisation, this can lead to the onset of life-threatening pathologies such as high-altitude pulmonary oedema (HAPE). The pathogenesis of HAPE remains unknown, and early diagnosis or prognostic prediction is essential for preventing morbidity, yet is not straightforward [56,57]. Metabolic analysis of plasma taken from 10 subjects at 3,658 m with HAPE revealed significant changes in 11 metabolites compared with healthy controls exposed to the same altitude; these include glycine (HMDB00123), citric acid (HMDB00094) and creatinine (HMDB00562) [58]. This study highlights the potential for the use of metabolomics as a diagnostic technique through the determination of disease biomarkers, although (as with many metabolomic biomarkers) these are likely to suffer from a lack of specificity. This has already been recognised for cardiovascular disease, and the current available biomarkers have the same limitations for screening purposes [37].

#### Metabolomics and the response to heat and cold

Many human populations live in extreme heat or cold. Both of these environmental stresses can cause exacerbation of disease and are potentially lethal [59,60]. The metabolic responses to neither extreme heat nor cold have been well investigated.

Extreme heat would be expected to induce an increase in metabolic processes and initiate heat-dissipating processes such as sweating. Although, to our knowledge, no metabolomics studies have been conducted in humans, the subject has been explored in a limited way in insects and rats. Results from these studies indicate shifts in TCA cycle functioning and an increased reliance on glycolysis during heat stress [61-63]. Inter-species differences obviously limit the translation of these results to humans, and work is required to further our understanding of the metabolome-wide response to heat stress in humans.

Acclimatisation to extreme cold undoubtedly relies heavily on behavioural adaptations such as the wearing of warm clothing and, in the short-term, shivering. However, with acclimatisation, there is also a heavy reliance on metabolic processes for non-shivering thermogenesis. Of particular interest are those changes taking place in mitochondria located within adipose tissue. Uncoupling of oxidative phosphorylation here is thought to play an essential role in cold-induced thermogenesis, a response believed to be regulated by the cold-sensing receptor TRPM8 expressed in both white and brown adipose tissue [64,65].

A strong correlation between basal metabolic rate (BMR) and climate has also been observed [66]. The BMR of indigenous Siberian populations, for example, was found to be 5% higher than values predicted based on body mass. These elevations appeared to be attributable to environmental stress rather than high dietary protein consumption, as was previously believed [67].

The reliance on metabolic processes for enabling survival in the extremes of heat and cold imply that the metabolic profile would undergo significant changes and so warrants further investigation.

#### Metabolomics and human spaceflight

A growing number of humans have experienced perhaps the most extreme of environments through spaceflight, and our understanding of the physiological response to an extended exposure to microgravity is similarly growing [68]. With commercial spaceflight, a realistic prospect for the next decade, this experience will be offered for the first time to individuals outside a highly trained elite corps of astronauts. It has been proposed that -omics methodologies, including metabolomics, form the cornerstone of a personalised medicine regime for the identification and treatment of microgravity-related conditions [69]. The application of metabolomics to head-down-tilt bedrest studies (as employed by NASA and ESA as a human spaceflight analogue) would be revealing in this regard, not least because of the documented effects of standard bedrest on exercise capacity, muscle wasting and insulin resistance, even in healthy young men [70].

## Conclusions

**Figure 1:**
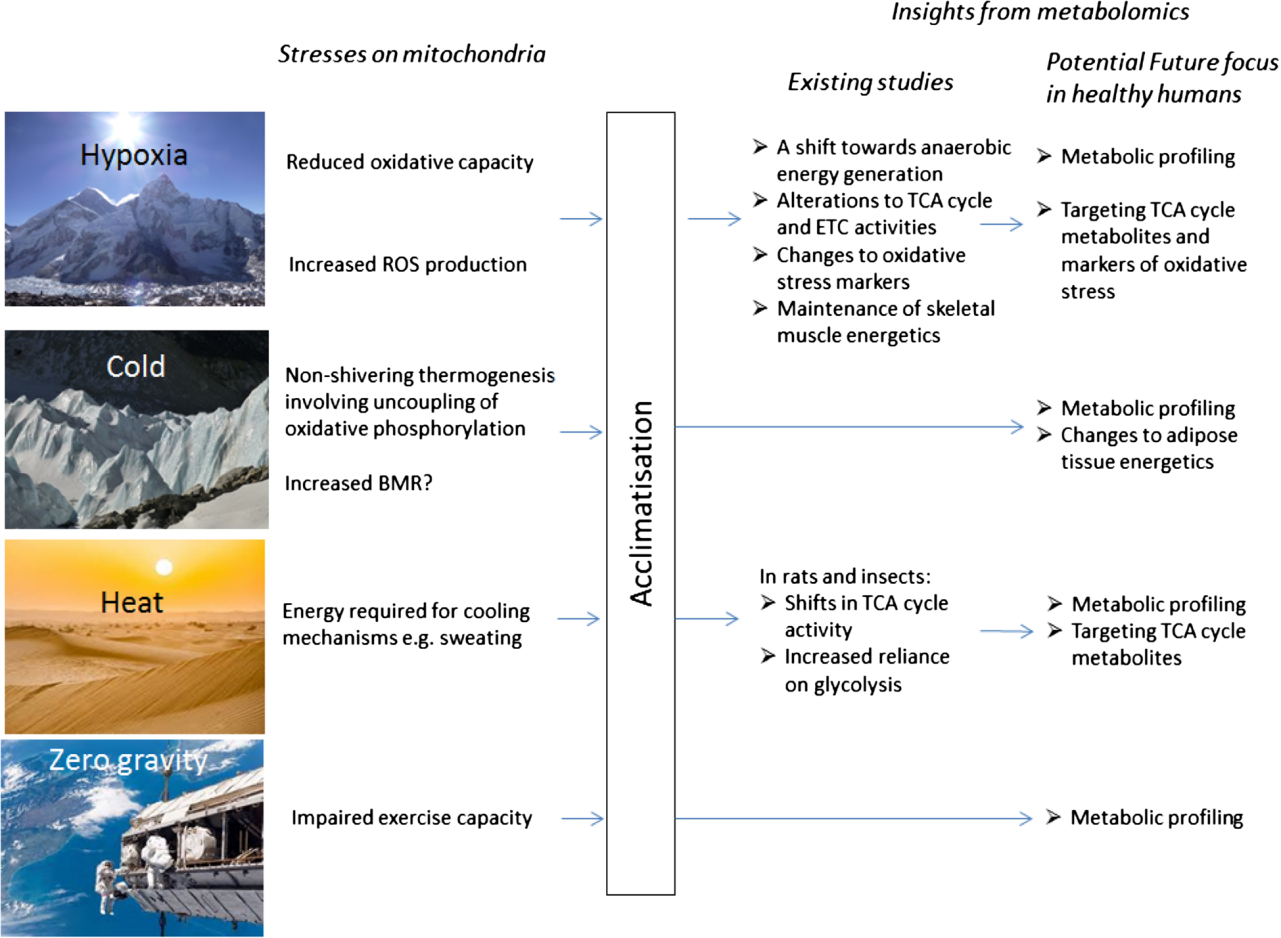
A summary of current knowledge on the stresses experienced by mitochondria in response to extreme environment exposure with insights into mitochondrial acclimatisation provided by existing metabolomics studies and suggested avenues of future metabolomics research.

It is clear that metabolomic studies of the human responses to altered climate and environment are worthwhile and lacking (summarised in Figure 1). Such studies could greatly further our understanding of human physiology and molecular biology. This work has the potential also to further our understanding of diseases that result from, or are akin to, physiology in extreme environments. It is clear that large-scale, longitudinal studies are required as studies conducted over longer periods of time would be better suited to providing information specific to these states. A combined use of targeted and untargeted approaches could also be employed to enable optimal detection.

## Abbreviations

TCA: Tricarboxylic acid cycle
NMR: Nuclear magnetic resonance
MS: Mass spectrometry
HR-MAS: High-resolution magic angle spinning
LC-MS: Liquid chromatography-mass spectrometry
GC-MS: Gas chromatography-mass spectrometry
ETC.: Electron transport chain
PCr: Phosphocreatine
HAPE: High-altitude pulmonary oedema
BMR: Basal metabolic rate
